# Longitudinal Evaluation of Otoacoustic Emissions as a Screening Tool for High‐Frequency Hearing Loss in Adolescents

**DOI:** 10.1002/ohn.70284

**Published:** 2026-05-20

**Authors:** Stefanie N. H. Reijers, Angeline E. Meerkerk‐Meijer, Jantien L. Vroegop, Geert Geleijnse, Bernd Kremer, Marc P. van der Schroeff

**Affiliations:** ^1^ Department of Otorhinolaryngology and Head and Neck Surgery Erasmus University Medical Center Rotterdam The Netherlands; ^2^ The Generation R Study Group Erasmus University Medical Center Rotterdam The Netherlands

**Keywords:** adolescents, high‐frequency hearing loss, otoacoustic emissions

## Abstract

**Objective:**

To investigate the association between distortion product otoacoustic emission (DPOAE) signal‐to‐noise ratios (SNRs) and pure‐tone audiometry (PTA) thresholds in adolescents, both cross‐sectionally at age 18 and longitudinally by evaluating whether DPOAE SNRs at age 13 predict high‐frequency hearing loss (HFHL) at age 18.

**Study Design:**

Prospective cohort study.

**Setting:**

The Generation R Study, a population‐based birth cohort in Rotterdam, the Netherlands.

**Methods:**

PTA thresholds and DPOAEs were obtained at ages 13 (April 2016 to September 2019) and 18 (October 2020 to May 2024). Participants with abnormal middle ear function, based on tympanometry, were excluded. HFHL was defined as PTA thresholds ≤ 15 dB HL at 500 and 1000 Hz and >15 dB HL averaged across 3, 4, 6, and 8 kHz. Receiver operating characteristic (ROC) curves were used to evaluate the ability of DPOAE SNRs to distinguish between HFHL and normal hearing. Sensitivity and specificity were calculated for different SNR thresholds.

**Results:**

Cross‐sectional analysis of 3463 ears at age 18 showed that DPOAE SNRs discriminated between ears with and without HFHL, yielding an area under the curve (AUC) of 0.830. Longitudinally, SNR values at age 13 predicted HFHL at age 18, with an AUC of 0.743.

**Conclusion:**

DPOAE SNRs demonstrate good accuracy for detecting HFHL in adolescents and moderate accuracy for predicting future HFHL. These findings support the potential role of DPOAEs as a noninvasive screening tool to enable early detection of high‐frequency cochlear dysfunction suggestive of early‐stage noise‐related hearing damage in youth.

Otoacoustic emissions (OAEs), first described by Kemp (1978), are low‐level sounds generated by the cochlea in response to auditory stimuli and may serve as a promising tool for detecting early cochlear damage.^1^ These emissions result from the active micromechanical processes of outer hair cells (OHCs) and can be measured in the external auditory canal using a sensitive microphone. OAEs reflect cochlear function independently of auditory nerve function objectively and noninvasively.^2^ While the absence of OAEs typically indicates OHC damage, their presence does not always distinguish between normal hearing and mild hearing loss.^3^

Of the various types of OAE, distortion product otoacoustic emissions (DPOAEs) are particularly sensitive to high‐frequency hearing loss (HFHL), which is often associated with noise‐induced hearing loss (NIHL).^3^, ^4^, ^5^ However, DPOAE amplitudes do not exhibit a direct one‐to‐one correspondence with pure‐tone thresholds. This discrepancy arises from interindividual variability and the complex interplay between nonlinear distortion and reflection components in OAE generation.^6^, ^7^ Additionally, OAEs can be affected by external factors such as noise, cerumen, and middle ear conditions.^8^ Understanding these limitations is essential to accurately evaluate the potential of DPOAEs as a predictive and diagnostic tool for HFHL.

Despite these challenges, OAEs offer the potential for early detection of subclinical cochlear dysfunction. Screening programs primarily rely on PTA, which detects hearing loss only after threshold shifts have become measurable—that is, once the cochlear damage has progressed to a clinically significant level.^9^, ^10^ In contrast, OAEs may detect early signs of cochlear damage before such changes are evident on PTA, thus improving opportunities for early intervention.^11^

This is particularly relevant in adolescence, a developmental stage where exposure to recreational noise—via personal audio devices, concerts, and other loud environments—is increasingly common.^12^, ^13^, ^14^, ^15^ Early cochlear damage during adolescence may have long‐term consequences on communication, academic performance, and mental health.^16^, ^17^, ^18^ Early detection is therefore crucial to reduce these long‐term effects and allow timely intervention.^12^, ^19^

While cross‐sectional studies have explored the association between OAEs and hearing thresholds, longitudinal data on their predictive validity remain limited.^3^, ^4^ Previous studies attempting to predict pure‐tone audiometry (PTA) thresholds based on DPOAEs have had limited success, particularly at higher frequencies or in cases of mild to moderate hearing loss, highlighting the complexity of their relationship.^20^, ^21^

This study aims to address this gap by examining the longitudinal relationship between DPOAE signal‐to‐noise ratios (SNRs) and high‐frequency pure‐tone thresholds in adolescents aged 13 to 18. Specifically, we will investigate whether DPOAE SNR values at age 13 can predict HFHL at age 18, and evaluate the diagnostic utility of SNRs at age 18 for detecting HFHL. By exploring the predictive value of DPOAEs, this research contributes to the development of more effective screening strategies for hearing loss in adolescents.

## Methods

### Study Design and Population

This study is part of the Generation R Study, a population‐based prospective cohort initiated from fetal life onward in Rotterdam, the Netherlands. The study protocol was approved by the Medical Ethics Committee of the Erasmus Medical Center, and written informed consent was obtained from the parents or legal guardians of all participating adolescents. Study details, including response rates and follow‐up procedures, have been published previously.^4^, ^22^, ^23^ Adolescents aged 13 to 18 years were invited to the Erasmus Medical Center – Sophia Children's Hospital for repeated hearing assessments at two time points: baseline (13 years) and follow‐up (18 years). Data collection occurred between April 2016 and September 2019 for the first measurement, and between October 2020 and June 2024 for the second measurement. Participants were eligible if they completed OAE testing, PTA, and had a normal tympanogram at both time points.

### Tympanometry

Middle ear function was assessed using an Interacoustics AT235h middle‐ear analyzer with a 226‐Hz probe tone. Criteria for exclusion included ear canal volumes < 0.3 mL, compliance < 0.25 mL, and/or middle ear pressure below −100 da Pa, which are indicative of potential middle ear pathology.^24^ Tympanometry results were measured at both time points to ensure reliable DPOAE recordings.^25^

### Distortion Product Otoacoustic Emissions

DPOAEs were recorded binaurally using an ILO292 Echoport USB‐II and UGD DPOAE + TEOAE probes with disposable tips. Weekly probe calibration and functionality checks were conducted, and proper probe fit was verified before each measurement. Emissions were elicited using two simultaneously presented primary tones (f1 and f2) at fixed levels of L1 = 65 dB SPL and L2 = 55 dB SPL, with a frequency ratio (f2/f1) of 1.22 to optimize distortion product amplitudes.^4^, ^26^, ^27^ Cubic distortion products (2f1‐f2) were measured at four points per octave across f2 frequencies ranging from 814 to 8000 Hz. Data collection at each frequency continued cyclic down until the noise floor dropped below −20 dB SPL or for a maximum of 90 seconds. For each f2 frequency, the SNR was calculated by subtracting the noise floor from the DPOAE level (in dB SPL). SNR was used as the primary outcome measure for predicting HFHL, as it reflects both the strength of the cochlear response and recording quality. The SNR was chosen over DPOAE amplitude alone because it accounts for background noise and provides a more robust and reliable estimate of cochlear function. To ensure reliable data, ears were excluded if the average noise floor across all frequencies exceeded the cohort mean plus two standard deviations.^28^ Additional exclusions were made in cases of poor recording conditions or uncertainty regarding probe placement.

### Pure‐Tone Audiometry

PTA was conducted in a sound‐treated booth that adhered to the maximum allowable ambient noise levels specified by ISO standard 8253‐1. Hearing thresholds were measured using a clinical audiometer (Decos audiology workstation, version 210.2.6 with AudioNigma interface) paired with TDH‐39P headphones and MX‐41/AR ear cushions. Air conduction thresholds were determined at 0.5, 1, 2, 3, 4, 6, and 8 kHz. Bone conduction thresholds were not measured due to time limitations. Testing started at 20 dB HL, with the sound level decreased by 10 dB or increased by 5 dB depending on whether a response was given by the participant. The hearing threshold was confirmed after at least two consistent responses out of three trials. Testing began with either the right or left ear, determined randomly.

### Statistical Analysis

Scatterplots were created to explore the relationship between pure‐tone thresholds and DPOAE levels at individual frequencies ranging from 3 to 8 kHz, which are primarily affected by noise exposure. Since the audiometric and f2 frequencies did not always align, we interpolated the DPOAE level at 3000 Hz using values from 2828 and 3364 Hz, and at 6000 Hz from 5657 and 6757 Hz. Additionally, the frequencies between 3 and 8 kHz were averaged, as demonstrated by Sisto et al (2007), who showed that averaging across a sufficient bandwidth improves the correlation between DPOAE levels and pure‐tone thresholds.^29^ A receiver operating characteristic (ROC) curve was generated to assess the diagnostic utility of the SNR in identifying HFHL. In this ROC analysis, the true positive rate (proportion of abnormal hearing ears correctly classified) was plotted against the false‐positive rate (proportion of normal hearing ears incorrectly classified as abnormal). The SNR, calculated from the DPOAE levels across frequencies from 2828 to 8000 Hz, was used to detect HFHL, as measured by PTA. HFHL was defined as (1) a threshold of 15 dB HL or less at 0.5 and 1 kHz, and (2) an average threshold of 3, 4, 6, and 8 kHz >15 dB HL. The area under the curve (AUC), the positive predictive value (PPV) and the negative predictive value (NPV) were used as the overall performance measure for the test. Statistical analyses were conducted using MATLAB R2024b.

## Results

At 13 years, a total of 3030 ears were included, with an equal distribution between right and left ears. The mean age was 13 years and 8 months old (SD ± 5 months), and 1773 (51.3%) were girls. At 18 years, a total of 3463 ears were included (49.8% right ears, and 50.2% left ears). The mean age was 18 years and 6 months (SD 8 months), and 1063 (53.8%) were girls.

### Pure‐Tone Audiometry

The mean hearing thresholds based on the PTA of the right and left ears are shown in Supplemental Figure S1, available online, for age 13 and in Supplemental Figure S2, available online, for age 18. At age 18, two different subgroups of participants with PTA were considered: (1) participants with a complete pure‐tone audiogram at age 18, a type A tympanogram at both age 13 and 18, and a complete DPOAE‐gram at age 13; and (2) participants with a complete pure‐tone audiogram at age 18, a type A tympanogram at age 18, and a complete DPOAE‐gram at age 18. In Group 1, 118 ears (3.9%) had HFHL, and in Group 2, 137 ears (4.0%) had HFHL.

### Distortion Product Otoacoustic Emissions

At age 13, average DPOAE levels ranged from −7.6 to 8.1 dB SPL across f2 frequencies between 841 and 8000 Hz, with peak responses observed at 1682 and 4757 Hz (Supplemental Figure S3, available online). Standard deviations indicated a high degree of variability, ranging from 9.6 to 12.8 dB SPL.

At age 18, DPOAE amplitudes ranged from −5.1 to 10.0 dB SPL, with the highest values observed at 1414 and 5657 Hz (Supplemental Figure S4, available online). As at age 13, variability was substantial, with standard deviations ranging from 8.4 to 11.2 dB SPL. After excluding ears with elevated hearing thresholds, mean levels ranged from −3.6 to 10.8 dB SPL.

In terms of SNR, the average SNR across all f2 frequencies was 12.1 dB SPL at age 13 and 12.9 dB SPL at age 18. At both ages, SNRs were generally highest in the mid‐frequency range and declined toward higher frequencies, consistent with reduced emission strength and increased background noise.

### Signal‐to‐Noise Ratio and High‐Frequency Hearing Loss

**Figure 1:**
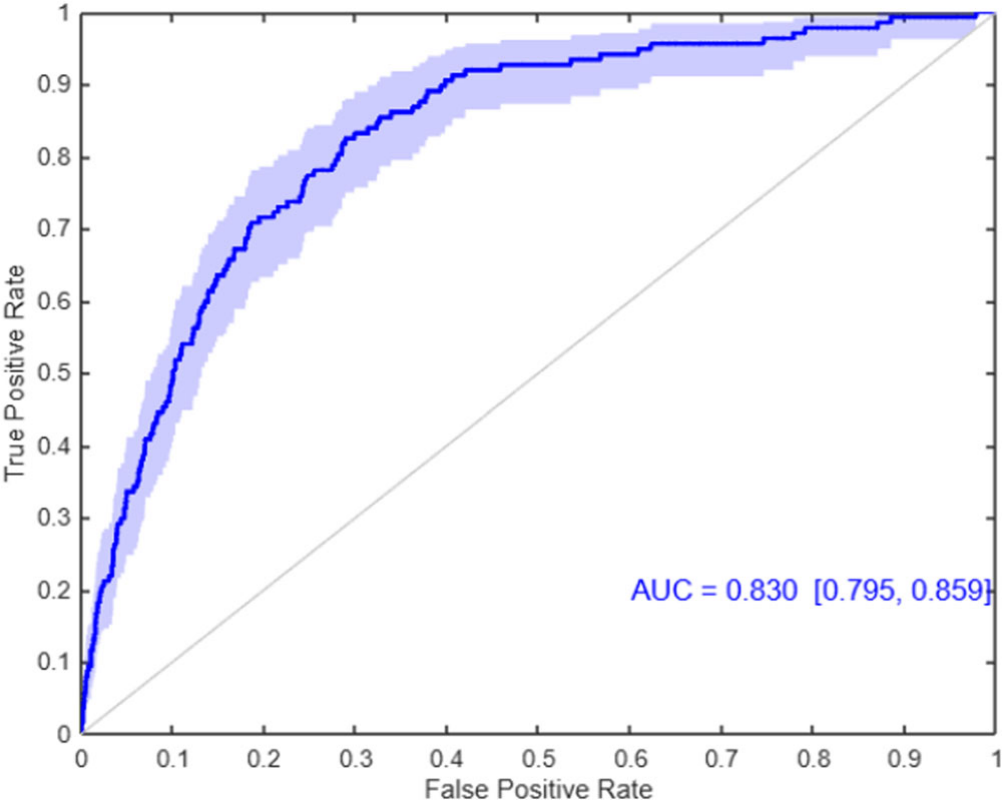
Cross‐sectional receiver operating characteristic (ROC) curve of signal‐to‐noise ratio (SNR) and high‐frequency hearing loss (HFHL) at age 18. ROC curve of SNR for HFHL with 95% confidence interval at 18 years. AUC, area under the curve.

**Figure 2:**
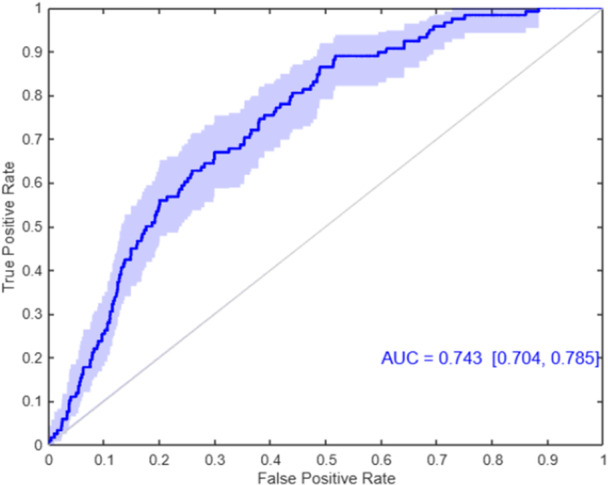
Longitudinal receiver operating characteristic (ROC) curve of signal‐to‐noise ratio (SNR) at age 13 and high‐frequency hearing loss (HFHL) at age 18. ROC curve of SNR for HFHL with 95% confidence interval at 13 and 18 years. AUC, area under the curve.

ROC curves were constructed to assess the ability of SNR values to differentiate ears with and without HFHL, based on SNR values averaged across f2 frequencies from 2828 to 8000 Hz. The cross‐sectional analysis at age 18 yielded an AUC of 0.830, and a PPV for a threshold SNR value of 6 dB is 0.16 with a NPV of 0.98. For the longitudinal analysis, SNR values at age 13 were used to predict HFHL at age 18. The corresponding ROC analysis resulted in an AUC of 0.743, and a PPV for a threshold SNR value of 6 dB is 0.10 with a NPV of 0.98. Figures 1 and 2 display the ROC curves for the cross‐sectional and longitudinal analyses, respectively. Table 1 presents the sensitivity and specificity of various SNR cutoff points for detecting HFHL at 18 years, Table 2 summarizes the longitudinal predictive value of SNR measurements from 13 to 18 years for HFHL at 18 years, and Table 3 shows the diagnostic accuracy of SNR values at 18 years for different definitions of HFHL.

**Table 1:** Sensitivity and Specificity of Signal‐to‐Noise Ratio (SNR) Values (2828‐8000 Hz) for Identifying High‐Frequency Hearing Loss: Cross‐Sectional (18 Years) and Longitudinal (13‐18 Years) Analyses

SNR value 2828‐8000 Hz (dB SPL)	Sensitivity (13 y.o.)	Specificity (13 y.o.)	Sensitivity (18 y.o.)	Specificity (18 y.o.)
−20	NaN	NaN	NaN	NaN
−15	0.00	1.00	NaN	NaN
−10	0.02	0.99	0.03	1.00
−5	0.14	0.95	0.14	0.98
0	0.24	0.91	0.30	0.96
5	0.45	0.84	0.48	0.90
10	0.64	0.71	0.72	0.80
15	0.86	0.51	0.92	0.56
20	0.98	0.24	0.96	0.26
25	1.00	0.05	0.99	0.06
30	1.00	0.00	1.00	0.00
35	NaN	NaN	NaN	NaN

**Table 2:** Predictive Accuracy of Signal‐to‐Noise Ratio (SNR) Values at 13 Years for Identifying High‐Frequency Hearing Loss at 18 Years

SNR 13 y.o., PTA 18 y.o.		
Definition of HFHL	Number of ears, %	AUC
3, 4, 6, and 8 kHz, >15 dB HL	118 (3.9)	0.743
3, 4, 6, and 8 kHz, >20 dB HL	41 (1.4)	0.762
3, 4, 6, and 8 kHz, >25 dB HL	18 (0.6)	0.821
4, 6, and 8 kHz, >15 dB HL	187 (6.2)	0.724
4, 6, and 8 kHz, >20 dB HL	51 (1.7)	0.794
4, 6, and 8 kHz, >25 dB HL	26 (0.9)	0.763

Abbreviations: AUC, area under the curve; HFHL, high‐frequency hearing loss; PTA, pure‐tone audiometry.

**Table 3:** The Accuracy With Which Signal‐to‐Noise Ratio (SNR) Value Can Identify Ears With High‐Frequency Hearing Loss Using Different Criteria of High‐Frequency Hearing Loss at 18 Years

SNR and PTA 18 y.o.		
Definition of HFHL	Number of ears, %	AUC
3, 4, 6, and 8 kHz, >15 dB HL	137 (4.0)	0.830
3, 4, 6, and 8 kHz, >20 dB HL	43 (1.2)	0.892
3, 4, 6, and 8 kHz, >25 dB HL	17 (0.5)	0.906
4, 6, and 8 kHz, >15 dB HL	210 (6.1)	0.797
4, 6, and 8 kHz, >20 dB HL	59 (1.7)	0.884
4, 6, and 8 kHz, >25 dB HL	27 (0.8)	0.901

Abbreviations: AUC, area under the curve; HFHL, high‐frequency hearing loss; PTA, pure‐tone audiometry.

### Sub‐analysis: Low Signal‐to‐Noise Ratio and No High‐Frequency Hearing Loss

At the age of 13, a total of 516 ears exhibited a low DPOAE SNR (<6 dB) at three or more frequencies ≥ 2000 Hz. Of these, 473 ears (92%) did not have HFHL when assessed using PTA. Follow‐up data at age 18 revealed that among these 473 ears, 22 (4.7%) met the criteria for HFHL.

Conversely, at age 13, 1415 ears demonstrated SNR values of 6 dB or more for at least seven frequencies at or above 2000 Hz. Among them, 1386 (98%) did not exhibit HFHL based on PTA assessment. By age 18, follow‐up data indicated that 19 of these 1386 (1.4%) fulfilled the criteria for HFHL.

## Discussion

This study is one of the first to investigate the relationship between DPOAE SNR values and HFHL in adolescents, combining both cross‐sectional and longitudinal analyses. Our findings suggest that DPOAE SNR values not only reflect current cochlear status but may also serve as early indicators of future hearing deterioration.

The role of DPOAEs in NIHL screening remains a subject of ongoing debate. Previous studies have demonstrated that DPOAEs can detect hearing losses between 20 and 30 dB HL.^30^, ^31^ Our study, however, focused on identifying early stages of hearing loss, which may not always fit into these traditional thresholds. In line with this, Attias et al (2001) found that DPOAEs can detect early NIHL even in individuals with normal audiograms but a history of noise exposure.^32^ In their study, NIHL was defined as a reduction in OAEs—particularly a notch at around 3 kHz in the DPOAE spectrum—relative to normative reference values, indicative of early cochlear damage despite normal behavioral thresholds. In our current study, DPOAEs were sensitive to detect HFHL at age 18. While the underlying cause of this HFHL cannot be confirmed, the audiometric pattern is suggestive of early‐stage NIHL. However, we acknowledge that HFHL can also result from other etiologies such as genetic predisposition, ototoxic medication, or other types of cochlear pathology. These findings nonetheless highlight the potential of DPOAEs to detect cochlear dysfunction before it becomes apparent in conventional audiometric tests.

In the longitudinal analysis, SNR values at age 13 showed moderate predictive ability for HFHL at age 18 (AUC = 0.743). Early DPOAE measurements had limited accuracy, reflected in a low PPV of 0.10 for the 6 dB threshold: 10% of those flagged “at risk” developed HFHL. This highlights the challenge of predicting future outcomes in a low‐prevalence population. However, the NPV was high (0.98), indicating that adolescents below the threshold were unlikely to develop HFHL. Clinically, individuals with reduced SNR might warrant closer monitoring or counseling. Predictive accuracy improved for more severe hearing loss (AUC = 0.821 for thresholds > 25 dB HL at 3‐8 kHz).

In the cross‐sectional analysis at age 18, the AUC of 0.830 indicates good overall discrimination, suggesting that SNR is a strong marker for HFHL at this age. The increased PPV of 0.16 demonstrates an increased accuracy in detecting HFHL at 18 with DPOAE. Despite good discrimination, the proportion of true positives among those classified as at risk remains low. The relatively low prevalence of HFHL in the population likely limits the PPV even when specificity and sensitivity are acceptable. Conversely, the NPV of 0.98 is very high, indicating that individuals below the threshold are very unlikely to have HFHL. The diagnostic ability to discern HFHL increases for more severe hearing loss.

These findings support the use of DPOAEs as an early warning tool for HFHL, including at subclinical thresholds. These results align with those of Portugal et al (2023), who found that early DPOAE measurements could predict future hearing impairment.^33^ It should be noted that, because HFHL is relatively rare and thresholds near 15 dB HL may be influenced by test‐retest variability, some caution is warranted when interpreting predictive accuracy of DPOAE measurements for milder HFHL. Additionally, our analysis revealed that DPOAEs were particularly effective in identifying more pronounced forms of HFHL, as evidenced by higher AUC values when stricter definitions of HFHL were applied. These findings are particularly relevant in adolescent populations, where age‐related changes in DPOAE levels have been observed.^34^ The higher AUC values for stricter HFHL thresholds suggest that DPOAEs may be particularly useful in identifying adolescents who are at risk of developing more clinically relevant or persistent HFHL, especially in the context of ongoing noise exposure. Depending on the goal of using DPOAEs within an early detection strategy, different SNR cutoff values may be appropriate—for example, lower thresholds for identifying individuals at increased risk of HFHL, and higher thresholds for ruling out HFHL in targeted follow‐up assessments.

Although DPOAEs are effective at identifying moderate to severe hearing loss, they are less sensitive when it comes to detecting mild hearing impairment. This variability is partly due to the complex nature of OAEs, which can be affected by factors such as ear canal resonance and middle ear function.^35^, ^36^, ^37^ Therefore, while DPOAEs are valuable as an early indicator of cochlear dysfunction, combining them with other diagnostic tools, such as PTA, could improve overall sensitivity and clinical reliability overall.

Taken together, these findings support the use of DPOAE SNR values as a noninvasive marker for both current and future HFHL in adolescents. In this context, “early intervention” should be interpreted primarily as targeted preventive counseling, increased awareness of safe listening behaviors, and closer audiological monitoring of adolescents at higher risk, rather than as interventions that differ fundamentally from general hearing conservation strategies applicable to all adolescents. It should be noted that the predictive performance reported in this study reflects the standalone value of DPOAE SNR measures and does not account for other factors known to influence hearing outcomes during adolescence, such as noise exposure, recreational listening habits, or genetic susceptibility. Because analyses were performed at the ear level, statistical non‐independence between ears from the same individual cannot be fully excluded and may have slightly inflated precision; future studies could address this by using subject‐level or mixed‐effects modeling approaches. Although this study did not directly assess individual noise exposure, future research should consider integrating detailed noise history or exposure measurements to better understand the pathways linking DPOAE reductions to hearing loss. Besides this tracking and analyzing the within‐subject longitudinal changes may offer complementary insights. Additionally, further work is needed to refine clinically relevant SNR cutoffs and evaluate the generalizability of findings across different adolescent populations. When considering implementing a DPOAE screening, the acoustics and settings of the screening environment should be similar to the controlled conditions in this current study, or a test‐retest study should be done to assess robustness in the desired screening environment. In addition to further investigating the use of DPOAEs for hearing screening in adolescents, a comprehensive cost‐benefit analysis should be conducted to evaluate the feasibility and desirability of implementing such a screening program.

## Conclusion

This study suggests that DPOAE SNR values may be useful in identifying HFHL in adolescents. The cross‐sectional analysis at age 18 showed a relatively high diagnostic accuracy (AUC = 0.830), and the longitudinal analysis indicated that DPOAEs at age 13 could have predictive value for HFHL at age 18 (AUC = 0.743). These findings point to the potential of DPOAEs as part of early detection strategies for HFHL in adolescents. Incorporating DPOAEs into adolescent hearing assessments could help identify individuals at risk of future hearing loss, though the PPV is moderate due to low prevalence. The high NPV shows that the DPOAE has an excellent potential as a secondary screening tool for ruling out HFHL in a target group. Further research is needed to confirm these results and clarify their clinical implications.

## Author Contributions

**Stefanie N. H. Reijers**: Study design; data acquisition; data analysis; drafting and revising the manuscript. **Angeline E. Meerkerk‐Meijer**: Study design; data acquisition; data analysis; drafting and revising the manuscript. **Jantien L. Vroegop**: Supervision; interpretation of findings; critical revision of the manuscript. **Geert Geleijnse**: Interpretation of findings; critical revision of the manuscript. **Bernd Kremer**: Supervision; critical revision of the manuscript. **Marc P. van der Schroeff**: Conceptualization; supervision; interpretation of findings; critical revision of the manuscript.

## Disclosures

### Competing interests

The authors declare no conflicts of interest.

### Funding source

This study was supported by the Netherlands Organization for Health Research and Development, Ministry of Health, Welfare and Sport, Netherlands Organization for Scientific Research (NWO), Erasmus University Rotterdam, and Erasmus Medical Center, Rotterdam.

## Supporting information


**Supplemental Figure S1. Mean hearing thresholds (dB HL) at 13 years**. Mean hearing thresholds (dB HL) of the right and left ears at 13 years.

**Supplemental Figure S2. Mean hearing thresholds (dB HL) at 18 years**. Mean hearing thresholds (dB HL) of the right and left ears at 18 years.

**Supplemental Figure S3. DPOAE and noise levels (dB SPL) at 13 years**. DPOAE and noise levels (dB SPL) at 13 years.

**Supplemental Figure S4. DPOAE and noise levels (dB SPL) at 18 years**. DPOAE and noise levels (dB SPL) at 18 years.
