# Supplementary figures and images for: Longitudinal Evaluation of Otoacoustic Emissions as a Screening Tool for High‐Frequency Hearing Loss in Adolescents
Source: Otolaryngol Head Neck Surg. 2026 May 20;175(2):480–6. doi: 10.1002/ohn.70284 (PMC13418088; doi:10.1002/ohn.70284)

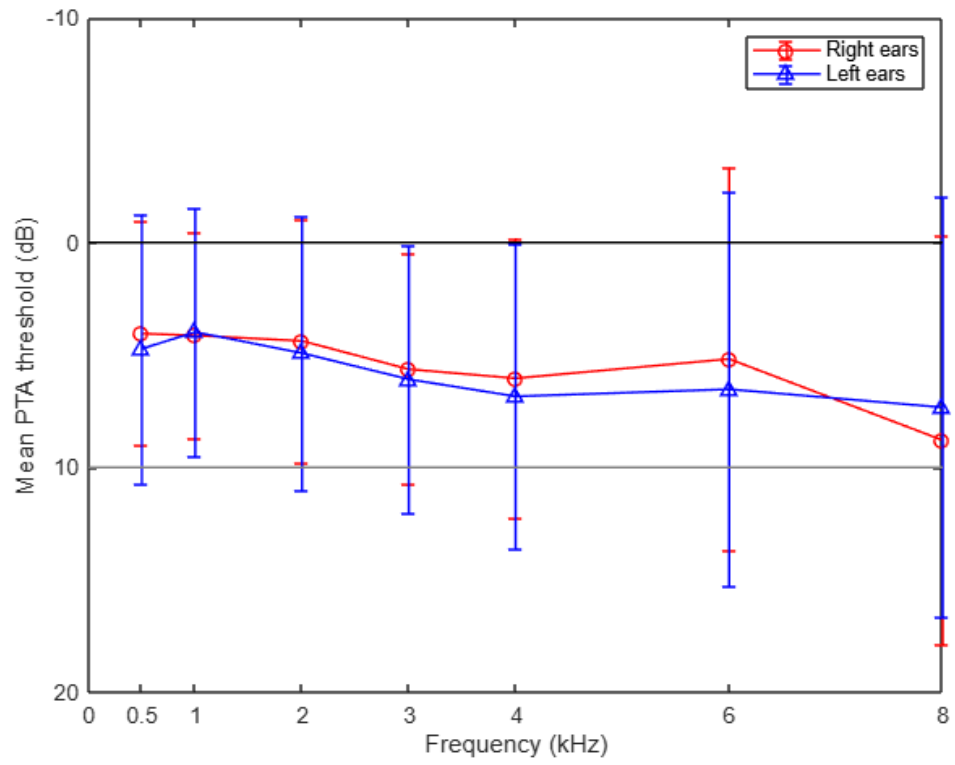

Supplement: Supplementary file 1 — Supplemental Figure S1. Mean hearing thresholds (dB HL) at 13 years. Mean hearing thresholds (dB HL) of the right and left ears at 13 years. [file OHN-175-480-s001.pdf]

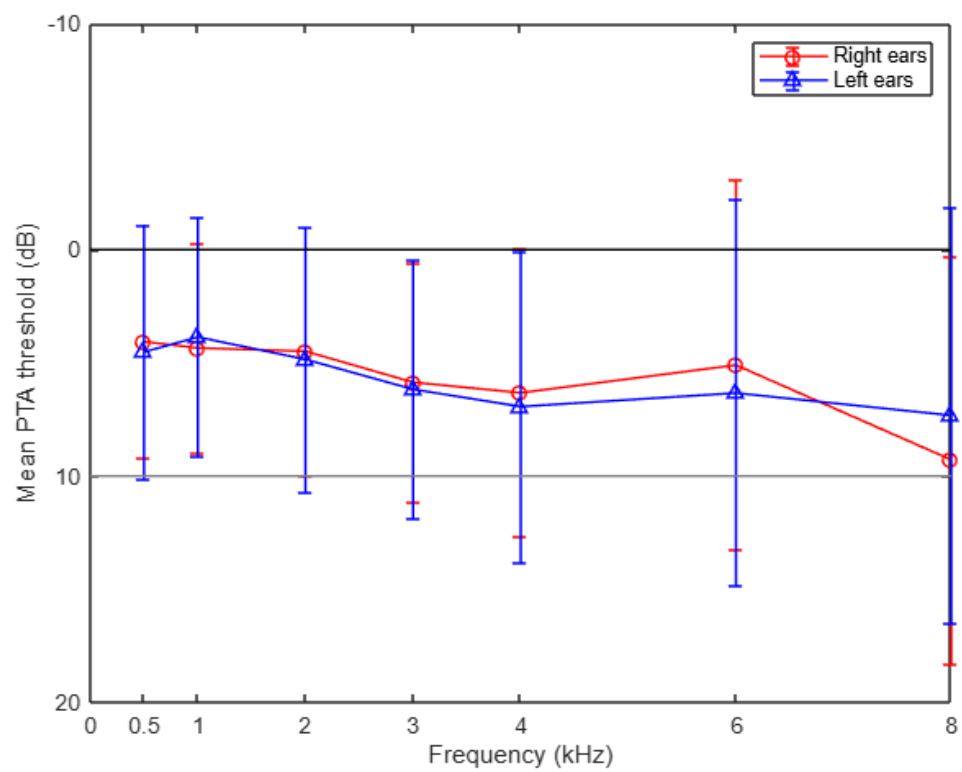

Supplement: Supplementary file 2 — Supplemental Figure S2. Mean hearing thresholds (dB HL) at 18 years. Mean hearing thresholds (dB HL) of the right and left ears at 18 years. [file OHN-175-480-s003.pdf]

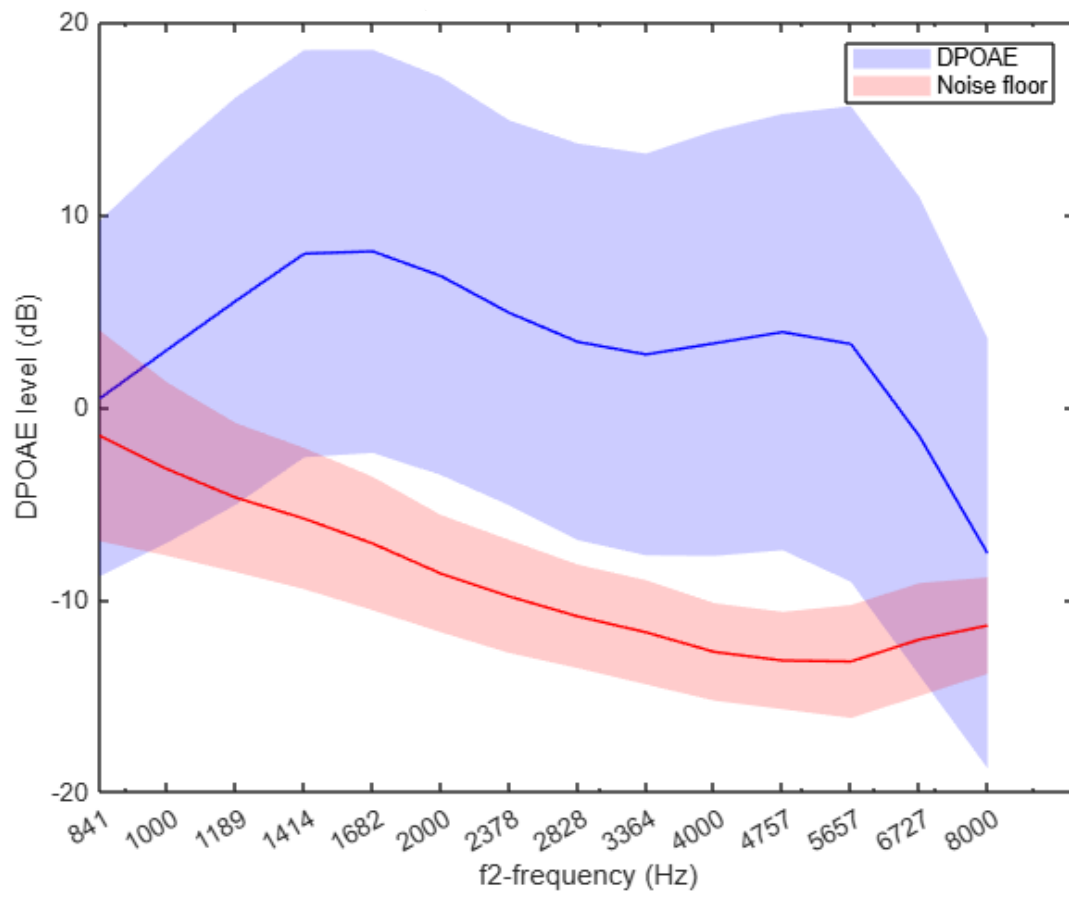

Supplement: Supplementary file 3 — Supplemental Figure S3. DPOAE and noise levels (dB SPL) at 13 years. DPOAE and noise levels (dB SPL) at 13 years. [file OHN-175-480-s002.pdf]

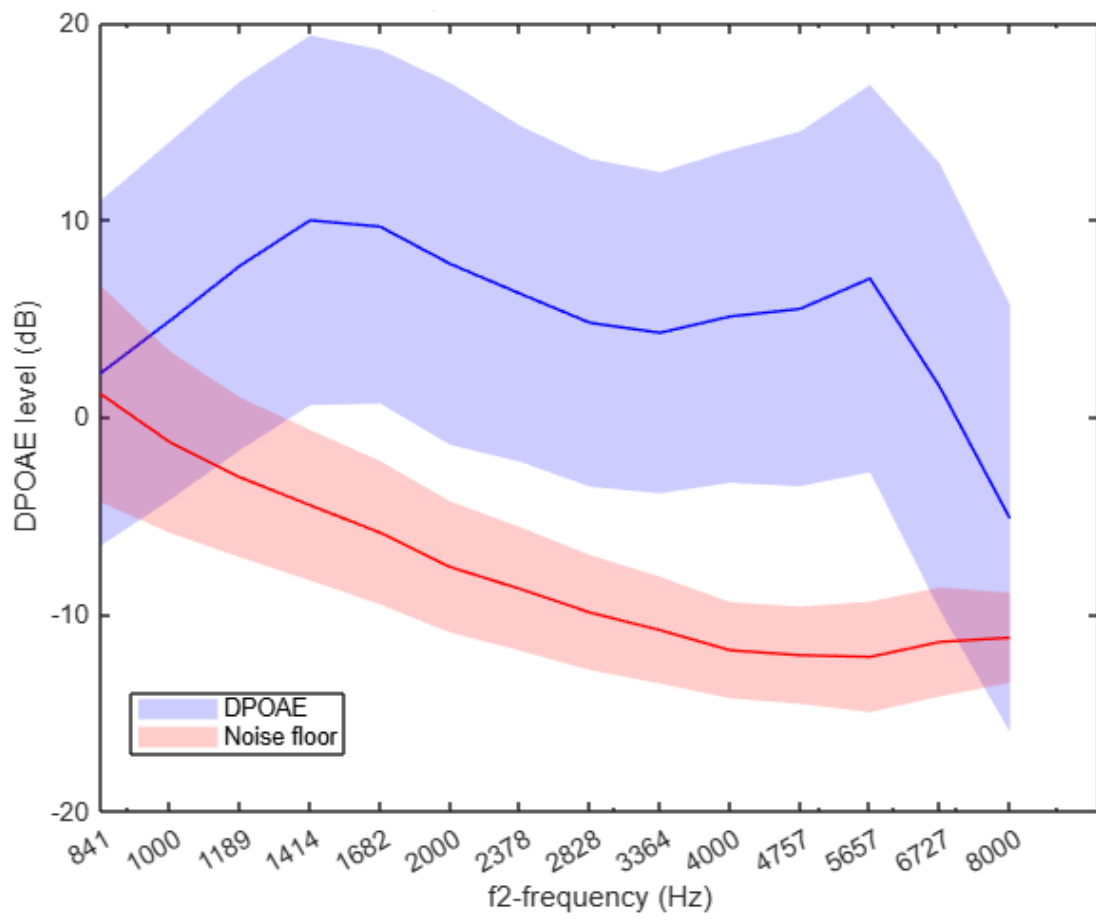

Supplement: Supplementary file 4 — Supplemental Figure S4. DPOAE and noise levels (dB SPL) at 18 years. DPOAE and noise levels (dB SPL) at 18 years. [file OHN-175-480-s004.pdf]
